# Wernekink commissure syndrome with palatal myoclonus at onset: a case report and review of the literature

**DOI:** 10.1186/s13256-018-1657-4

**Published:** 2018-05-01

**Authors:** Naresh Mullaguri, Anusha Battineni, Miguel Chuquilin

**Affiliations:** 1 Department of Neurology, University of Missouri, DC. 047, CE530, CS&E Building, 5 Hospital Drive, Columbia, MO 65212 USA; 2 Department of Neurology, University of Florida, HSC Box 100236, Gainesville, FL 32610 USA

**Keywords:** Wernekink commissure syndrome, Internuclear ophthalmoplegia, Palatal myoclonus, Guillain-Mollaret triangle, Ataxia, Dysarthria

## Abstract

**Background:**

Wernekink commissure syndrome causes a peculiar combination of internuclear ophthalmoplegia, dysarthria, and delayed-onset palatal myoclonus. Palatal myoclonus is thought to be secondary to delayed hypertrophic degeneration of the bilateral inferior olivary nuclei secondary to involvement of bilateral dentatoolivary tract. We describe a case of a patient with early-onset palatal myoclonus.

**Case presentation:**

A 53-year-old Caucasian man with several vascular risk factors presented to our emergency room with slurred speech, double vision, difficulty with swallowing and walking, and rhythmic contractions of the soft palate. Brain magnetic resonance imaging showed an acute infarct of the right caudal midbrain and an old infarct of the right medulla. We hypothesize that the cause of early palatal myoclonus in our patient was a two-hit mechanism with degeneration of the right olivary nucleus resulting from prior right medullary lacunar stroke with the new infarct affecting the dentato-rubro-olivary tract on the left side, causing bilateral dysfunction initiating palatal myoclonus.

**Conclusions:**

Wernekink commissure syndrome with palatal myoclonus at onset suggests the presence of a prior ischemic insult in the medulla. Careful examination is important to identification of this presentation.

## Background

Wernekink commissure syndrome is a rare midbrain syndrome characterized by bilateral cerebellar ataxia, internuclear ophthalmoplegia, dysarthria, and delayed-onset palatal myoclonus or Holmes tremor secondary to a stroke in the paramedian caudal midbrain region [1–4]. The midbrain derives most of its blood supply from the posterior cerebral arteries through penetrating perforators from the P1 segment and the basilar artery. Depending on the location and extent of infarction in the caudal midbrain, the clinical presentation can be variable. Because the medial midbrain is densely packed with structures such as oculomotor nuclei, descending cerebellar tracts, medial longitudinal fasciculus, and central tegmental tract, a lesion in this area can present with internuclear ophthalmoplegia on the affected side, unilateral or bilateral ataxia, dysarthria, dentatorubral tremor, and delayed-onset palatal myoclonus secondary to hypertrophic degeneration of bilateral inferior olivary nuclei [1–10]. Symptoms are mostly bilateral at presentation, because the paramedian caudal midbrain contains descending cerebellar tracts, central tegmental tract, and other important white matter tracts crossing the midline, making it difficult for the untrained eye to localize the lesion to one vascular territory.

To the best of our knowledge, cases of Wernekink commissure syndrome due to midbrain infarction presenting with internuclear ophthalmoplegia, ataxia, and palatal myoclonus at symptom onset have not been reported in the literature to date.

## Case presentation

A 53-year-old right-handed white man presented to the emergency room of our institution with a 2-day history of double vision, dysarthria, and difficulty with swallowing and walking. His symptoms were sudden in onset with gradual worsening. He could not walk owing to fear of falling and was unable to eat or drink because of choking. His double vision was worse with horizontal gaze to the left side. He noticed clumsiness and incoordination in both upper and lower extremities. A review of systems was unremarkable for headache, nausea, vomiting, fever, facial pain, new-onset weakness or numbness, neck pain or stiffness, or weight and appetite loss. The patient’s past medical history was significant for hypertension, type 2 diabetes mellitus, hyperlipidemia, obstructive sleep apnea, and right medullary infarction 2 years prior to presentation. He had residual left-sided weakness and ambulated with a cane at baseline. He denied smoking, drinking alcohol, and use of recreational drugs. His home medications included amlodipine, atenolol, clopidogrel, gabapentin, insulin, losartan, metformin, spironolactone, and ibuprofen.

At presentation, the patient’s systolic blood pressure was elevated in the range of 200–220 mmHg. His physical examination was significant for disconjugate eye movements with multidirectional nystagmus, right-sided medial rectus palsy on left-sided horizontal gaze suggestive of right-sided internuclear ophthalmoplegia (INO), no afferent pupillary defect, and a normal pupillary reflex and fundus examination. His facial sensations were normal to fine touch and painful stimuli. There was no facial muscle weakness. He had continuous, involuntary, and rhythmic contractions of the soft palate without an audible clicking sound. The results of the patient’s motor examination were significant for spasticity, mild weakness, and brisk deep tendon reflexes in the left upper and lower extremities. His sensations were diminished to vibration up to the ankles bilaterally with unremarkable fine touch and pain sensation. He had abnormal finger-to-nose and heel-to-shin test results in the upper and lower extremities bilaterally. He was unable to stand with his eyes open. No tremors were noticed in his head or upper and lower extremities. His National Institutes of Health Stroke Scale score was 6 (2 points each for dysarthria, ataxia, and left-sided upper and lower extremity drift). The differential diagnoses considered were possible multifocal infarction in the posterior circulation; demyelinating diseases such as acute demyelinating encephalomyelitis, multiple sclerosis, and neuromyelitis optica; and neuroinfectious diseases such as Whipple’s disease and neurosarcoidosis. Acute stroke was considered high in the differential diagnosis, given his multiple uncontrolled vascular risk factors, prior history of stroke, and the acuity of symptom onset.

**Fig. 1:**
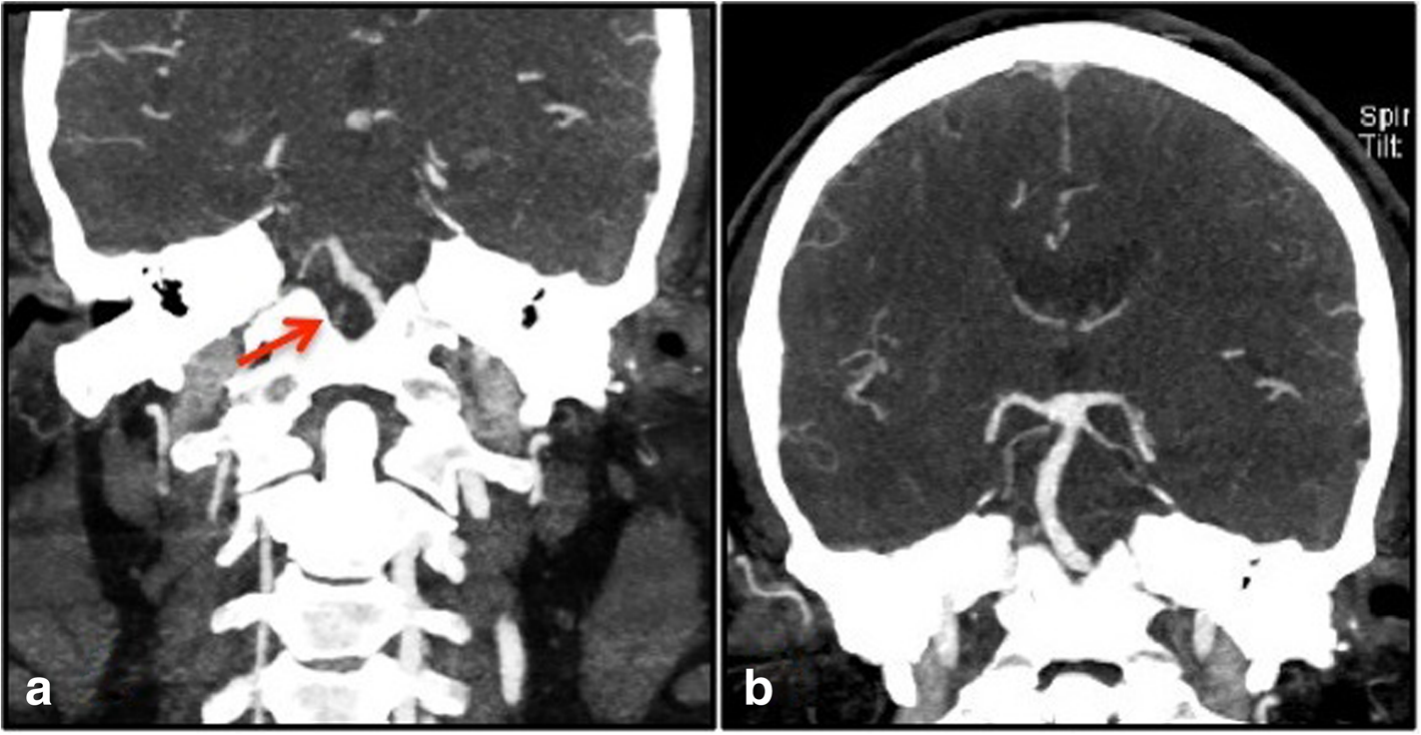
Computed tomographic angiogram of the head and neck coronal sections. **a** Severe atherosclerotic stenosis of the right vertebral artery at the V4 segment with complete occlusion prior to the formation of the basilar artery (*red arrow*). **b** Basilar tip sessile aneurysm giving off bilateral posterior cerebral and superior cerebellar arteries

**Fig. 2:**
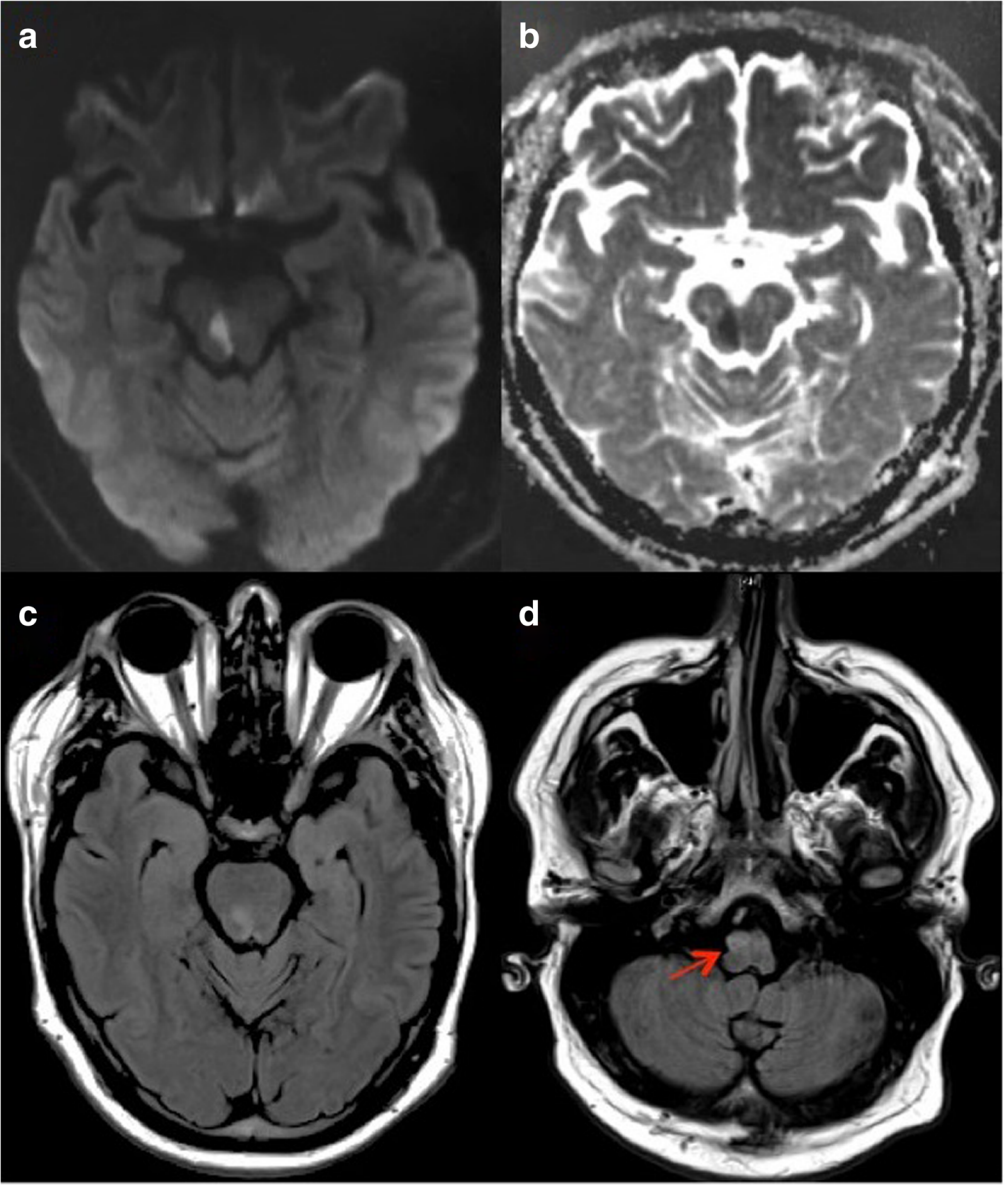
Brain magnetic resonance imaging axial sections. **a** and **b** Diffusion-weighted imaging and apparent diffusion coefficient sequences show diffusion restriction in the right caudal midbrain region. **c** and **d** T2-weighted fluid-attenuated inversion recovery sequences show hyperintensity in the right caudal midbrain with acute infarction and hypertrophic degeneration of the right olivary nucleus (*red arrow*)

The patient’s blood workup revealed leukocytosis of 18,900 cells/mm^3^ (reference range 3600–11,200 cells/mm^3^), which decreased to 14,800 cells/mm^3^ the next day. He had mild elevation of blood urea nitrogen at 27 mg/dl (reference range 9–25 mg/dl) and creatinine at 1.34 mg/dl (reference range 0.7–1.3 mg/dl) at admission, which were normalized the following day with intravenous hydration. We thought that the patient’s leukocytosis and slightly elevated renal parameters were due to dehydration. The result of his urine toxicology screen was unremarkable. A clopidogrel resistance panel showed a subtherapeutic response with adenosine diphosphate inhibition of 31% (reference range 50–100%). He had a glycated hemoglobin A1c of 7.6 g %, low-density lipoprotein level of 178 mg/dl, and triglyceride level of 359 mg/dl. A computed tomographic (CT) scan of the patient’s head showed mild burden of small vessel disease without hemorrhage or early signs of ischemic stroke. CT angiography of his head and neck showed chronic occlusion of the V4 segment of the right vertebral artery with distal reconstitution by collateral branches 5 mm before the origin of the basilar artery with a sessile aneurysm measuring 7 mm × 7 mm at the basilar tip (Fig. 1). On day 2 of the patient’s admission, magnetic resonance imaging of the brain showed a small area of restricted diffusion in the right caudal midbrain suggestive of acute lacunar infarct with additional findings of chronic infarction in the right caudal medulla and T2 hyperintensity with hypertrophy of the right inferior olivary nucleus (Fig. 2). His electrocardiogram showed sinus rhythm with no arrhythmias on continuous telemetric monitoring during hospitalization. A transthoracic echocardiogram showed normal ejection fraction, moderate concentric left ventricular hypertrophy along with diastolic dysfunction, and no right-to-left shunting. The etiology of stroke was possibly secondary to vessel-to-vessel thromboembolism resulting from severe intracranial atherosclerotic disease of the right vertebral artery. He was started on dual antiplatelet therapy with aspirin and clopidogrel. We also optimized his modifiable vascular risk factors by adjusting his antihypertensive and statin medications for secondary prevention of ischemic stroke. He was discharged to an inpatient rehabilitation center on day 3. The patient was lost to follow-up.

## Discussion

Our patient had a unique presentation of Wernekink commissure syndrome with tetra-ataxia, internuclear ophthalmoplegia, and palatal myoclonus at the onset of a caudal midbrain lacunar infarction. To the best of our knowledge, such a case has not been reported in the literature to date. Palatal myoclonus is known to occur as a late phenomenon due to hypertrophic degeneration of bilateral inferior olivary nuclei. It is not the case in our patient with prior right medullary infarction causing degeneration of the ipsilateral inferior olive with the new caudal midbrain infarction affecting the decussation of dentato-rubro-olivary tracts. This may have caused dysfunction of bilateral dentato-rubro-olivary tracts leading to palatal myoclonus at the onset of stroke.

Wernekink commissure syndrome was first described by Lhermitte in 1941 and first published in 1958. It was named after German anatomist Friedrich Wernekink, who gave the description of brachium conjunctivum as a horseshoe-shaped commissure [11]. The Wernekink commissure consists of two important white matter tracts: the ascending dentato-rubro-thalamic tract and the descending dentato-rubro-olivary tract. The ascending dentato-rubro-thalamic tract connects the dentate nucleus of the cerebellum through the superior cerebellar peduncle to the contralateral red nucleus and thalamus. The descending dentato-rubro-olivary tract connects dentate and interposed nuclei of the cerebellum with the contralateral red nucleus through the superior cerebellar peduncle and the inferior olivary nucleus in the medulla [12]. Lesions of the Wernekink commissure at the caudal midbrain may damage the dentato-rubro-thalamic or dentato-rubro-olivary pathways, which leads to early or delayed Holmes tremor and delayed-onset palatal myoclonus or tremor, respectively. The clinical presentation varies depending on the vascular distribution of midbrain arterial perforators that arise from the tip of the basilar artery, superior cerebellar arteries, and precommunicating P1 segment of posterior cerebral arteries called *interpeduncular fossa perforators* [13]. Owing to the proximity of the medial longitudinal fasciculus and trochlear nucleus to the brachium conjunctivum and dentate-rubro-olivary tracts, a caudal midbrain infarction either unilaterally or bilaterally can cause bilateral cerebellar ataxia, dysarthria, multidirectional nystagmus, unilateral or bilateral internuclear ophthalmoplegia, and variable eye movement disorders [1–10]. In contrast to the cases previously reported in the literature (Table 1) with palatal myoclonus occurring several months after ischemic stroke, our patient presented with palatal myoclonus from the onset. Matsuo *et al.* and Tuna *et al*., in their case reports, hypothesized that delayed palatal myoclonus was secondary to lesions involving the Guillain-Mollaret triangle leading to delayed transsynaptic degeneration of bilateral inferior olivary nuclei [7, 14, 15]. The onset of palatal myoclonus with acute stroke in our patient questions this hypothesis because there was no evidence of left inferior olivary nucleus degeneration seen on imaging (Fig. 1d, arrow). We hypothesize that the cause of early palatal myoclonus in our patient was due to a two-hit mechanism with degeneration of the right olivary nucleus from prior right medullary infarct and acute right caudal midbrain infarct affecting the dentato-rubro-olivary tract on the left side owing to interruption at the decussation, causing bilateral dysfunction and initiating the palatal myoclonus. As described by Bolen *et al.* and Mossuto-Agatiello *et al*., despite the degeneration of bilateral olivary nuclei, their patients did not develop palatal myoclonus, thus defining the heterogeneity of this syndrome and its unclear pathophysiology [1, 8]. Vascular neurologists should be aware of this unique midbrain lacunar stroke syndrome presenting with an interesting palatal movement disorder, which can be very disabling for the patient.

**Table 1:** Clinical and neuroradiological characteristics of paramedian caudal midbrain stroke cases reported in the literature

Case report	Age/sex	Vascular risk factors	Clinical presentation						Neuroimaging results		
			Eye movement disorder	Dysarthria	Ataxia	Tremor	Palatal myoclonus	Other	Infarction	Hemorrhage	HOD and laterality
Bolen *et al.* [1]	53/M	HTN, HLD, type 2 DM, ESRD, hemorrhagic stroke	Eight-and-a-half syndrome	–	–	+	+			Left pontine hemorrhage extending into midbrain	Left
Dai *et al.* [2]	70/M	HTN, DM, HLD, PVD	Left INO	+	+	–	–	Intermittent jaw clonus, visual and auditory hallucinations	Left caudal midbrain		–
Kim *et al*. [3]	62/M		Upbeat nystagmus	+	+	–	+		Caudal midbrain		–
Krespi *et al*. [4]	57/F		Right INO	–	+	–	–		Right paramedian caudal midbrain		–
Liu *et al.* [16]	a) 59/F	HTN	Right gaze-evoked horizontal nystagmus	+	+	–	+		Caudal midbrain		–
	b) 71/M	Stroke	Left INO	+	+	–	+	Right hemiparesis	Left paramedian caudal midbrain		–
Zhu, *et al.* [6]	59/F	HTN	Horizontal nystagmus	+	+	–	–	Dysmetria	Infarction in the central tegmentum of the midbrain at the level of superior cerebellar peduncle decussation		–
Sheetal *et al.* [17]	51/M	HTN, CAD	Right INO	+	+	–	–	–	Left paramedian midbrain lacunar		–
Zhou *et al.* [18]	60/M	HTN	Bilateral INO	+	Bilateral extremities and truncal ataxia	Head tremor	+	–	Bilateral caudal paramedian midbrain		Bilateral
Mossuto-Agatiello *et al.* [8]	a) 64/M	DM, HTN, MI	–	+	+	–			Right paramedian caudal midbrain		–
	b) 52/^a^	HTN	–	+	+	–	–		Left caudal paramedian midbrain		–
	c) 38/M		Horizontal nystagmus with skewed eyes, upward gaze restriction,inappropriate convergence	–	+	–	–	Headache, coma with incomplete locked-in syndrome	Basilar artery occlusion with infarction in superior cerebellar peduncles, cerebellar hemispheres and left thalamus (intracranial vertebral artery dissection was possible etiology)		–
	d) 34/F	Oral contraceptive pills, smoking	Right oculomotor palsy,INO, bilateral horizontal nystagmus	+	+	–			Right paramedian caudal midbrain		Bilateral
	e) 42/M	Basilar tip aneurysm with acute onset of symptom status postrepair	Non-pupil-sparing right oculomotor palsy, left-sided ptosis	+	+	–	–	Tetraparesis	Paramedian caudal midbrain		–
Menéndez *et al*. [9]	27/^a^	Right midbrain cavernous malformation	Double vision, right-sided ptosis	+	–	Delayed Holmes tremor	–	Left hemiparesis			Bilateral
Mullaguri, *et al.* (our patient)	53/M	HTN, type 2 DM, HLD, OSA, and previous right medullary infarction	Right INO	+	Tetra ataxia	–	+	–	Right caudal midbrain infarction, right vertebral artery atherosclerosis, and sessile aneurysm of the tip of the basilar artery		Right

*Abbreviations*: *HTN* Hypertension, *DM* Diabetes mellitus, *HLD* Hyperlipidemia, *PVD* Peripheral vascular disease, *MI* Myocardial infarction, *CAD* Coronary artery disease, *ESRD* End-stage renal disease, *INO* Internuclear ophthalmoplegia, *HOD* Hypertrophic Olivary Degeneration, *OSA* Obstructive Sleep Apnea

^a^Patient’s sex not reported

## Conclusions

Wernekink commissure syndrome is one of the rare paramedian caudal midbrain stroke syndromes that can cause tetra-ataxia, internuclear ophthalmoplegia, dysarthria, variable eye movement disorders, Holmes tremor, and delayed palatal myoclonus. Palatal myoclonus as a presenting symptom at stroke onset is rare and might suggest prior brainstem ischemic lesions causing hypertrophic olivary nucleus degeneration.

## Abbreviations

CAD: Coronary artery disease
CT: Computerized tomography
DM: Diabetes mellitus
ESRD: End-stage renal disease
HLD: Hyperlipidemia
HTN: Hypertension
INO: Internuclear ophthalmoplegia
MI: Myocardial infarction
MRI: Magnetic resonance imaging
PVD: Peripheral vascular disease
